# Genetics and Pathogenesis of Parkinson’s Syndrome

**DOI:** 10.1146/annurev-pathmechdis-031521-034145

**Published:** 2022-09-13

**Authors:** Hui Ye, Laurie A. Robak, Meigen Yu, Matthew Cykowski, Joshua M. Shulman

**Affiliations:** 1 Department of Neurology, Baylor College of Medicine, Houston, Texas, USA;; 2 Jan and Dan Duncan Neurological Research Institute, Texas Children’s Hospital, Houston, Texas, USA; 3 Department of Molecular and Human Genetics, Baylor College of Medicine, Houston, Texas, USA;; 4 Department of Neuroscience, Baylor College of Medicine, Houston, Texas, USA;; 5 Department of Pathology and Genomic Medicine, Houston Methodist Hospital, Houston, Texas, USA;; 6 Department of Neurology, Houston Methodist Hospital, Houston, Texas, USA; 7 Center for Alzheimer’s and Neurodegenerative Diseases, Baylor College of Medicine, Houston, Texas, USA

**Keywords:** Parkinson’s disease, parkinsonism, oligogenic, functional genomics, heterogeneity, *GBA*, *LRRK2*, alpha-synuclein, synapse, lysosome, mitochondria

## Abstract

Parkinson’s disease (PD) is clinically, pathologically, and genetically heterogeneous, resisting distillation to a single, cohesive disorder. Instead, each affected individual develops a virtually unique form of Parkinson’s syndrome. Clinical manifestations consist of variable motor and nonmotor features, and myriad overlaps are recognized with other neurodegenerative conditions. Although most commonly characterized by alpha-synuclein protein pathology throughout the central and peripheral nervous systems, the distribution varies and other pathologies commonly modify PD or trigger similar manifestations. Nearly all PD is genetically influenced. More than 100 genes or genetic loci have been identified, and most cases likely arise from interactions among many common and rare genetic variants. Despite its complex architecture, insights from experimental genetic dissection coalesce to reveal unifying biological themes, including synaptic, lysosomal, mitochondrial, and immune-mediated mechanisms of pathogenesis. This emerging understanding of Parkinson’s syndrome, coupled with advances in biomarkers and targeted therapies, presages successful precision medicine strategies.

## INTRODUCTION

More than 200 years following its clinical recognition and 100 years after its pathologic description, a comprehensive understanding of Parkinson’s disease (PD) remains elusive. PD is a neurodegenerative condition that has traditionally been recognized clinically by a distinctive motor phenotype (parkinsonism) and pathologically by the presence of Lewy bodies and substantia nigra neuronal loss. However, many of the central tenets that have classically defined the disorder have been recently challenged. For example, though first described for its motor manifestations, PD nonmotor features are now also widely acknowledged and represent a major factor in disease-related disability. Similarly, whereas the discovery of Lewy body pathology, comprising alpha-synuclein (αSyn) protein inclusions, identified a major disease trigger, some PD cases lack Lewy bodies, and other brain pathologies also have important roles. In short, the more we learn about PD and its heterogeneity, the more difficult it becomes to categorize it as a single disease on the basis of strict clinical and pathologic criteria. Instead, the emerging picture is more befitting of a syndrome—a fragmented collection of many different conditions with variable clinical and/or pathologic overlap (1, 2). The goal of this review is to describe the rapidly expanding understanding of PD genetics in the context of this growing recognition of the Parkinson’s syndrome. At first glance, the genetic architecture of PD seems to compound the complexity—hundreds of genes, abundant allelic heterogeneity, incomplete penetrance, and vast potential for gene–gene and gene–environment interactions. However, we argue instead for an emerging biological consensus. Underlying the surface-level complexity, our functional genomic understanding of PD reveals crosscutting, shared disease mechanisms that will soon change clinical practice for diagnosis and management.

## CLINICAL CONTEXT

The cardinal motor features of PD—bradykinesia (slowed movements), rigidity (increased muscle tone), tremor, and altered gait and postural reflexes—collectively compose a pattern of impairment termed parkinsonism (3). PD is the most important cause of parkinsonism and the second most common neurodegenerative disorder after Alzheimer’s disease. In fact, PD prevalence is increasing more rapidly than virtually any other neurologic condition, with more than 6 million cases estimated globally (4). PD diagnosis is determined on the basis of a careful medical history and neurological examination, requiring the essential presence of bradykinesia along with rigidity and/or rest tremor. Additional weighting of positive, supportive features (e.g., response to dopaminergic therapy) or negative features (e.g., signs of cerebellar dysfunction) are used to increase diagnostic confidence and to rule out potential alternatives. Ancillary testing is usually not required to establish a PD diagnosis, but may be helpful in certain situations. For example, brain magnetic resonance imaging is sometimes useful to exclude less common and atypical causes of parkinsonism. Similarly, dopamine transporter single-photon emission computed tomography may reveal dopaminergic insufficiency in individuals with mild parkinsonism. Indeed, PD develops insidiously, with a decades-long clinical prodrome consisting of mild and often unrecognized symptoms (5).

PD clinical presentations are remarkably heterogeneous (6, 7). While aging is an important risk factor, with a peak prevalence of ~85 years, PD can be diagnosed over a wide age range. Young-onset PD, arbitrarily defined on the basis of manifestation of disease before ~45 years, accounts for a small minority of cases. PD can also vary in the presence and severity of specific motor features. For example, tremor is absent in approximately a third of cases. PD clinical heterogeneity has long spurred efforts to define phenotypic subtypes (8). Tremor- versus gait-predominant forms are recognized, and these distinct motor signatures associate with other pertinent features of disease progression, complications, and disability. PD subtyping is further complicated by changing disease manifestations over time. For example, gait is frequently spared early but compromised in more advanced disease. PD nonmotor manifestations are ubiquitous, but these also vary in their presence, severity, and/or timing (9). In fact, nonmotor features, including reduced olfaction and sleep dysregulation [rapid eye movement sleep behavior disorder (RBD)], frequently precede the development of motor manifestations (5, 10). On the basis of prospective cohort studies, ~80% of individuals with RBD will ultimately develop PD or a related neurodegenerative disorder (11). Whereas dopaminergic and other available therapies are highly effective for PD motor impairments, nonmotor manifestations are comparatively refractory to treatment and are therefore a major contributor to disability in PD (12). Dysfunction of the autonomic nervous system in PD causes constipation, dizziness due to blood pressure fluctuations, and reduced bladder control. Neuropsychiatric manifestations are also common, including anxiety, depression, psychosis, and dementia (13, 14). Mild cognitive impairment can also accompany prodromal PD and is present in up to 20% of patients at the time of diagnosis, particularly executive dysfunction (e.g., deficits in planning, working memory, mental flexibility) (13). Cumulatively, 80% of individuals with PD develop dementia within two decades (12). Using unsupervised clustering and considering both motor and nonmotor features in an untreated PD sample, three major PD clinical subtypes were identified: mild motor predominant, intermediate, and diffuse malignant (15). The latter group, comprising 12% of the study cohort, was characterized by greater motor disability, increased nonmotor features (reduced olfaction, autonomic dysfunction, and cognitive impairment), and rapid disease progression.

On the basis of large case series with autopsy confirmation, the clinical diagnostic accuracy of PD is only 80% (16). Indeed, parkinsonism is also caused by several other neurodegenerative and nonneurodegenerative conditions, which can be challenging to differentiate from PD clinically, especially early in the disease course. Among these atypical parkinsonian neurodegenerative disorders, progressive supranuclear palsy is characterized by abnormal eye movements as well as gait and postural impairment, leading to earlier falls than usually seen with PD (17). Corticobasal syndrome is differentiated by markedly asymmetric parkinsonism in association with cortical sensory deficits and/or inability to perform skilled movements (apraxia). Multiple system atrophy causes parkinsonism along with cerebellar and autonomic dysfunction (18). When dementia precedes or develops within one year of motor symptom onset, the alternate clinical diagnosis of dementia with Lewy bodies should be entertained (13, 19). However, PD dementia and dementia with Lewy bodies are pathologically indistinguishable (see below), and the umbrella term Lewy body dementia is now commonly used for both of these clinical syndromes. Parkinsonism can also be caused by cerebrovascular disease (e.g., vascular parkinsonism), medications (e.g., antipsychotics), toxins (e.g., manganese, carbon monoxide), or it can even follow viral infection (e.g., postencephalitic parkinsonism) (6, 7).

## PATHOPHYSIOLOGY

Parkinsonism arises primarily from dysfunction of the basal ganglia, an interconnected group of subcortical and brainstem nuclei with a major role in regulating the initiation and smooth execution of movements. Specifically, the loss of dopaminergic neurons in the midbrain substantia nigra compromises signaling to the striatum (caudate plus globus pallidus), leading to nigrostriatal insufficiency. In animal models, lesioning or optogenetic manipulations causing similar perturbations recapitulate the cardinal motor features of PD (20–22). Dopamine transporter imaging of the human brain can also reveal PD basal ganglia pathophysiology, and restoration of circuit function is the basis for neurosurgical therapies, such as deep brain stimulation. Evidence from both neuroimaging and clinicopathologic studies strongly suggest that the loss of synapses and/or axonal degeneration contribute to initial PD motor manifestations, even preceding neuronal loss (23). Reserve and compensatory mechanisms also play a major role. For example, it is estimated that at the time of PD diagnosis, 30–50% of dopaminergic neurons are already lost, and an even greater proportion of dopaminergic terminals may be dysfunctional (24, 25). Moreover, not all midbrain dopaminergic neurons are equally vulnerable to degeneration in PD, with those occupying the ventrolateral tier being particularly susceptible and others, such as those of the ventral tegmental area, being less affected. Critically, other neuronal subtypes are also affected in PD, and distinct neurotransmitter systems likely underlie the heterogeneous manifestations of Parkinson’s syndrome (1). In fact, neurodegeneration in the locus coeruleus and dorsal raphe frequently precedes pathologic change within the substantia nigra (26), and the resulting dysfunction in noradrenergic and serotonergic pathways contribute to PD nonmotor manifestations, such as depression and fatigue (9). Disruption of cholinergic pathways arising from the basal forebrain is implicated in PD dementia. Interestingly, neurons vulnerable to degeneration in PD may share a distinctive structural and physiologic signature, with diffuse axonal arborization and tonic firing patterns (27).

**Figure 1:**
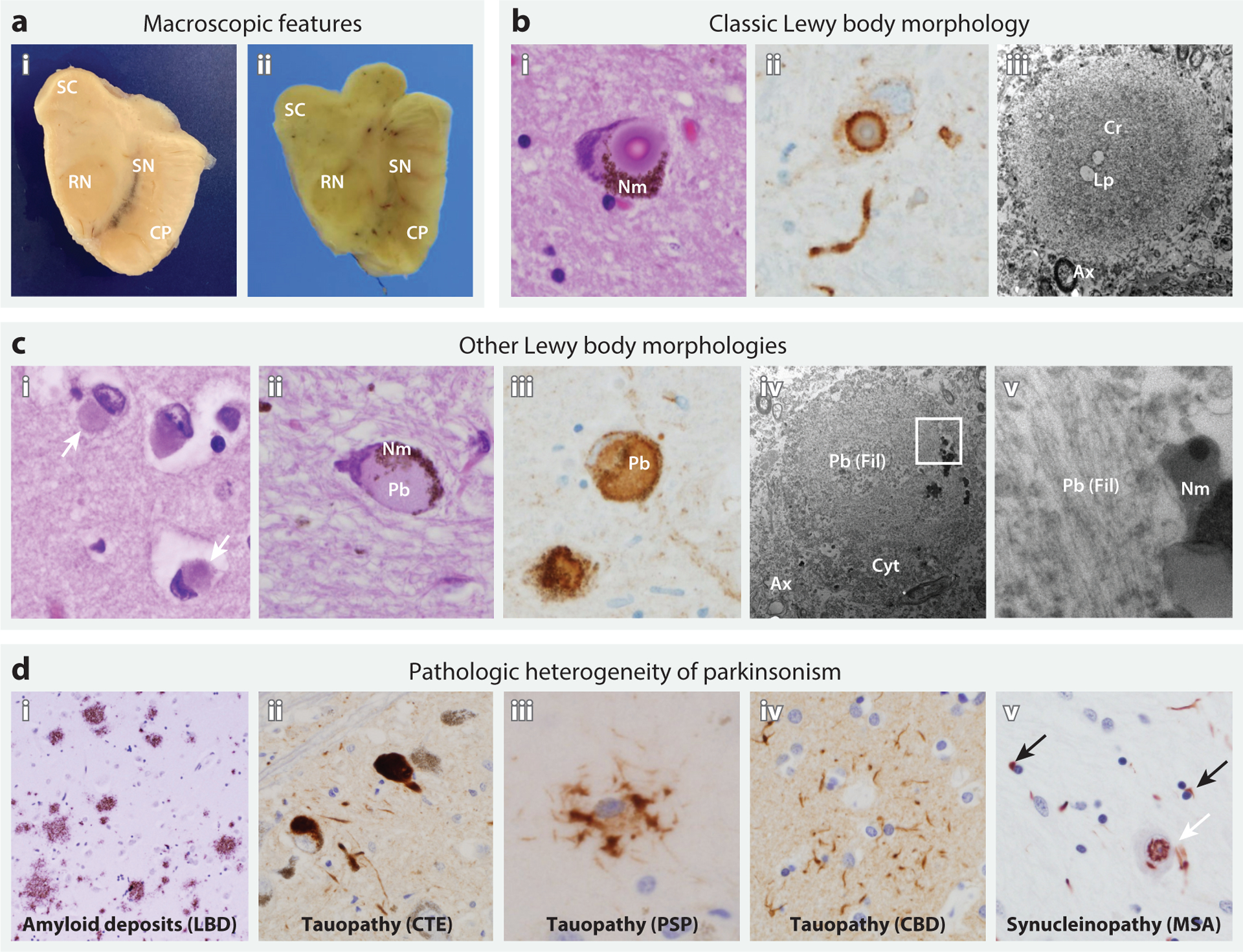
Neuropathologic features of Lewy body diseases (*a*,*b*,*c*) and other causes of parkinsonism (*d*). (*a*) Macroscopic appearance of the midbrain in (*i*) a patient without parkinsonism and (*ii*) a patient with Lewy body dementia (LBD), highlighting the superior colliculus (SC), red nucleus (RN), cerebral peduncle (CP), and substantia nigra (SN). In LBD, SN pigmentation is lost, whereas tectal (SC) and tegmental (RN) volumes are unaffected. Relative preservation of midbrain volume helps distinguish PD from mimics, such as progressive supranuclear palsy (PSP). (*b*) Classic Lewy body morphology in the SN on the basis of microscopic examination, including (*i*) a cytoplasmic inclusion with a dense eosinophilic core, concentric lamellae, and a pale-staining peripheral rim (hematoxylin and eosin stain). The Lewy body displaces neuromelanin (Nm) pigment within the cytoplasm. (*ii*) An immunostain for alpha-synuclein (αSyn) shows the dense staining of the peripheral rim of an inclusion, with less dense staining of the core. The tortuous, αSyn-positive structure below is a Lewy neurite. (*iii*) Electron microscopy of an extracellular Lewy body in the SN shows the characteristic dense core (Cr), here with sparse lipid droplets (Lp), a looser filamentous rim, and the adjacent neuropil with axons (Ax). (*c*) Other Lewy body morphologies, including (*i*) cortical Lewy bodies (*white arrows*) from temporal neocortex and (*ii*) pale bodies (Pb) from SN. (*iii*) An immunostain shows diffuse αSyn labeling of a Pb. (*iv*,*v*) Electron microscopy reveals noncompact, granulofilamentous material (Fil), entrapping and displacing cytoplasmic organelles (Cyt), and the adjacent neuropil with axons (Ax). The region enclosed by the white square is magnified (*v*), showing filamentous material that is sharply demarcated by Nm granules. (*d*) Pathologic heterogeneity of parkinsonism. Diffuse amyloid plaques are shown from a patient with LBD (*i*), along with characteristic pathology from several tauopathies with prominent parkinsonism, including (*ii*) tau-positive SN neuronal inclusions in chronic traumatic encephalopathy (CTE), (*iii*) a p62-positive tufted astrocyte from the striatum in PSP, and (*iv*) a tau-positive astrocytic plaque from the frontal cortex in corticobasal degeneration (CBD). (*v*) Multiple system atrophy (MSA) is characterized by αSyn-positive oligodendroglial cytoplasmic inclusions (*black arrows*); however, other pleomorphic, including intranuclear, inclusions are also commonly seen in neurons (*white arrow*).

Besides dopaminergic neuronal loss, the pathologic diagnosis of PD requires the presence of αSyn immunoreactive cytoplasmic inclusions in neurons, termed Lewy bodies (28, 29) (Figure 1). αSyn is an abundant, 140-amino-acid neuronal protein that is enriched at synapses and likely participates in neurotransmission (30). Normally, αSyn exists in an equilibrium between a soluble unstructured state and a lipid-bound conformation consisting of amphipathic alpha-helical repeats. However, αSyn undergoes extensive posttranslational modifications in PD, including phosphorylation (e.g., Ser129), and assumes a toxic beta-sheet conformation capable of aggregation. While αSyn is a major constituent of Lewy bodies, hundreds of other proteins are also present, and recent ultrastructural analysis highlights abundant lipid membranes and dysmorphic organelles, including lysosomes and mitochondria (31). αSyn pathology arises from a complex cascade starting with the formation of soluble, oligomeric species and leading to assembly into amyloid fibrils and other higher-order, insoluble structures (32). Substantial evidence now supports a model in which pathologic forms of αSyn, also called strains, can seed the misfolding of endogenous αSyn, acting as a template to amplify and even propagate pathology (33). αSyn misfolding and pathology appear highly context dependent. In multiple system atrophy, which is characterized by glial cytoplasmic inclusions and a more aggressive clinical course (18), the oligodendrocyte cellular milieu appears capable of inducing αSyn to assume a distinct and more potent, toxic strain conformation (34, 35). Similarly, prion-like properties have also been demonstrated for other neurodegenerative triggers, such as β-amyloid and tau in Alzheimer’s disease, and this behavior may contribute to the transmission and spread of pathology throughout the nervous system (36).

Elegant clinicopathologic studies have elucidated the patterns and likely progression of αSyn pathology in the nervous system (26, 37, 38). On the basis of the Braak staging criteria, PD starts either centrally, in the olfactory bulb, or peripherally, in the enteric nervous system (stage 1), before ascending the brainstem and ultimately involving limbic and neocortical regions (stage 6), with sparing of the motor cortex. In this scheme, Lewy body pathology in the midbrain substantia nigra is not seen until stage 3, subsequent to involvement of lower brainstem regions, such as the dorsal motor nucleus of the vagus nerve and reticular formation in the medulla. Indeed, αSyn pathology may be widespread in the peripheral and autonomic nervous system, potentially permitting early detection from gastrointestinal or skin biopsies (39, 40). These observations have led to the provocative hypothesis that αSyn pathology may spread transsynaptically, at least in part, along neuroanatomic circuits. While this model remains controversial, the propagation of αSyn pathology from gut to brain via the vagus nerve has been demonstrated in mouse models (41). Indirect support for this model comes from epidemiology suggestive of reduced PD risk among individuals with a history of vagus nerve transection (42). Importantly, a substantial minority of PD cases—nearly half in some series—appear to diverge from the bottom-up predictions of the Braak staging scheme; alternatively, pathology appears initially in brain limbic regions and/or the olfactory bulb with secondary, top-down spread to the brainstem (43–46). The alternate body-first versus brain-first PD pathologic patterns have been recapitulated in vivo using a suite of complementary neuroimaging modalities to assess involvement of peripheral autonomic system and both lower and upper brainstem pathways (47). Compounding the complexity, accumulating evidence suggests that substantia nigra neuronal loss can precede the detection of Lewy bodies, suggesting that other forms of αSyn may be more likely directly responsible for mediating tissue injury (48, 49).

Beyond αSyn pathology, other brain lesions contribute significantly to PD pathogenesis. Most Lewy body dementia (>80%) and a substantial proportion of all PD cases (>30%) have comorbid Alzheimer’s disease pathologic change, including β-amyloid deposits (neuritic and diffuse plaques) and tau-positive neurofibrillary tangles (46, 50, 51). TAR DNA-binding protein 43 (TDP-43) inclusion pathology, which is characteristic of frontotemporal lobar degeneration and amyotrophic lateral sclerosis, is also commonly found (52). Reciprocally, αSyn pathology is frequently comorbid in those with Alzheimer’s disease, increasing susceptibility for dementia (53). Evidence from mouse models, along with in vitro studies, highlights how pathologic forms of αSyn, β-amyloid, and tau can interact with one another, possibly enhancing aggregation and neurotoxicity (32, 54–56). Together, these findings support a model in which Alzheimer’s pathology may accelerate the deposition and/or spread of αSyn pathology within limbic and neocortical regions of the brain, perhaps promoting the diffuse malignant subtype of PD and/or dementia with Lewy bodies. Mixed pathologies likely also contribute to the development of PD motor manifestations, including cerebrovascular lesions (e.g., microscopic infarcts and arteriosclerosis) (57). Pathologic heterogeneity is further underscored by autopsy studies of Mendelian forms of PD (e.g., *PRKN*, *VPS35*), in which nigral degeneration can occur in the absence of Lewy body pathology or following other variable postmortem findings (58). Indeed, besides Lewy bodies, other neuropathologies are important drivers of atypical parkinsonian syndromes, as in the tauopathy known as progressive supranuclear palsy (17).

## GENETICS

For nearly as long as we have recognized PD, there has been ongoing debate about risk factors, including the relative contributions of nature versus nurture. Besides age, epidemiologic studies have identified male sex, pesticide exposure, and traumatic brain injury as PD risk factors (59). Among potential protective factors, physical activity, smoking, and caffeine exposure have the most robust evidence. The role of genetic risk in PD was controversial until relatively recently, with positive family history even considered exclusionary in past diagnostic criteria. Nevertheless, beginning with the landmark 1997 discovery of mutations in *synuclein-alpha* (*SNCA*) (60), the last 25 years have witnessed remarkable progress, with ~100 distinct genes or loci having now been definitively linked to PD susceptibility (61). Approximately 15–25% of PD patients report a history of other affected family members, and recent meta-analyses suggest a greater than fourfold increased risk for PD among such individuals (62, 63). Moreover, genome-wide genotyping in large cohorts has enabled estimation of PD heritability, with up to 36% of overall disease risk due to common genetic variation (64). On many levels, the rapid pace of genetic discovery challenges the conceptualization of PD as a single disease entity. We organize our discussion of PD genetics around several overarching questions.

### What Is the Difference Between Familial and Sporadic PD?

**Figure 2:**
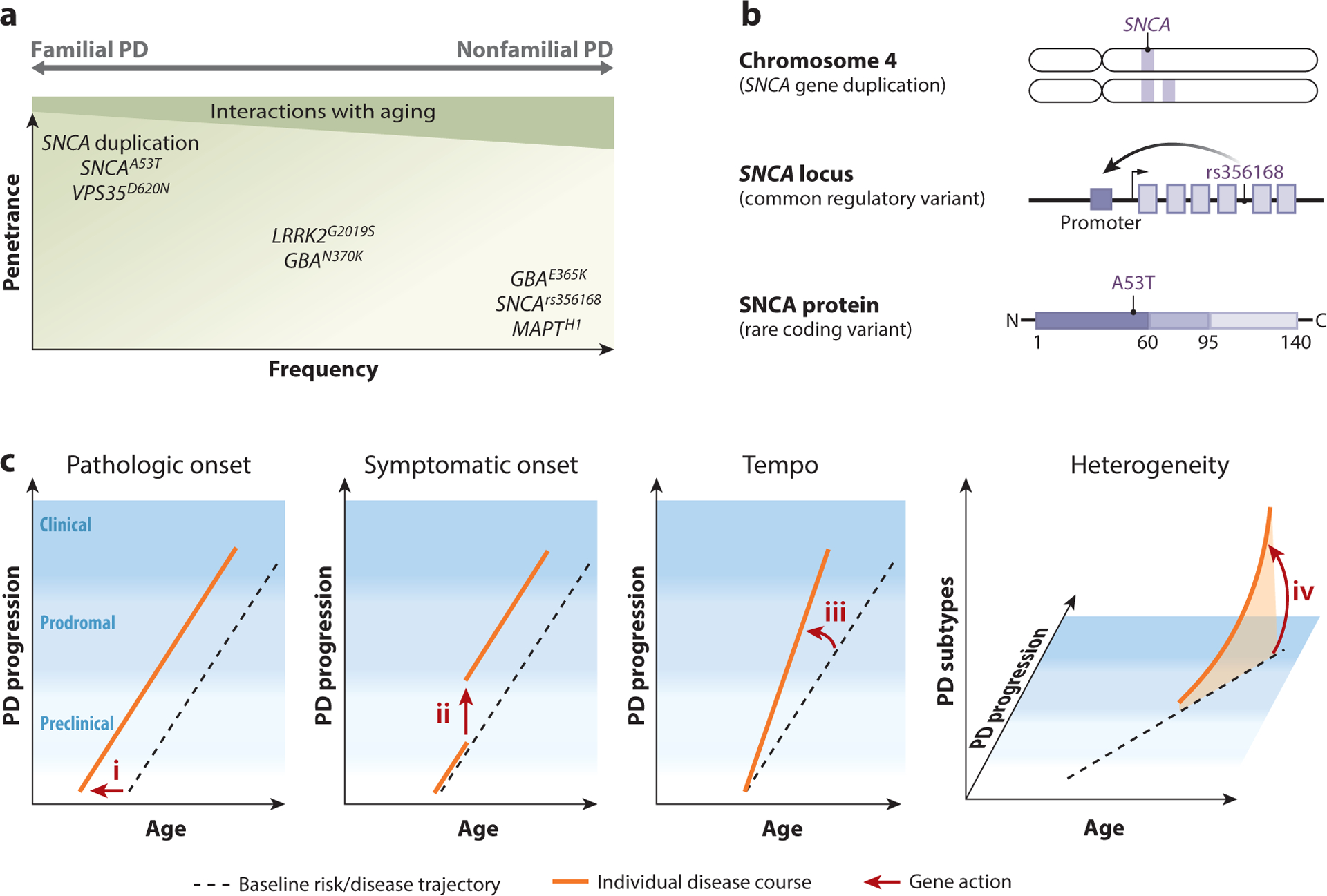
Genetic architecture of Parkinson’s disease (PD). (*a*) PD risk alleles have a wide spectrum of frequencies and effect sizes (penetrance). Many PD risk alleles also show age-dependent penetrance. The manifestation of familial versus nonfamilial PD depends on allele penetrance, age, and the presence of genetic and environmental modifiers. (*b*) Allelic heterogeneity of *synuclein-alpha* (*SNCA*). Copy number variants (duplication) and both common (rs356168) and rare (p.A53T) single-nucleotide variants affecting *SNCA* are associated with PD risk. Locus duplication and common regulatory variants that enhance gene promoter activity increase overall gene expression levels. Rare variants promote SNCA protein aggregation. (*c*) Conceptual model for how PD genetic variants (*arrows i*–*iv*) might affect risk and overall course of disease (*orange lines*). The black dotted line represents the baseline population PD risk and disease trajectory. Genetic risk factors might influence (*i*) the earliest formation and propagation of disease pathology or (*ii*) the conversion from preclinical to prodromal disease (first clinical symptoms). It is also possible that genes modify (*iii*) the overall tempo of PD clinicopathologic progression. Beyond PD initiation and progression, genetic factors might plausibly impact (*iv*) the heterogeneous manifestations of PD, such as the presence and severity of specific motor and nonmotor features.

The term sporadic PD is frequently used to refer to cases without a family history and is often differentiated from familial PD. However, given our current understanding of PD genetic architecture, these labels can be confusing and somewhat misleading. Specifically, the distinction between familial and nonfamilial PD may be misinterpreted to imply that there exists a subset of cases in which genetic mechanisms do not apply. However, we now recognize that virtually all PD cases likely have detectable genetic influences, with the specific genetic variant(s) involved in individual cases varying by frequency and effect size (Figure 2a). Familial PD, also commonly referred to as Mendelian or monogenic PD, is generally recognized when rare, high-penetrance variants influence disease risk. Both autosomal dominant (e.g., *SNCA*^*A53T*^, *VPS35*^*D620N*^) and autosomal recessive (e.g., *PRKN*, *PINK1*) forms exist (61). Such genetic variants were initially discovered using linkage analysis in families, and more recently next-generation sequencing methods have facilitated additional discoveries. Overall, single-gene variants, including high-penetrance, Mendelian alleles, are discovered in 5–10% of all PD cases (63). On the other hand, common genetic variants with low-penetrance effects are more frequently associated with sporadic PD, and such variants have largely been discovered through genome-wide association studies (GWAS) (61, 64). Differentiating familial from sporadic disease—or genes with high- versus low-penetrance risk alleles—may have meaningful implications in clinical practice, such as for diagnosis, prognosis, and genetic counseling of at-risk family members. However, this classification also risks obscuring shared genetic and biological mechanisms. For example, both rare and common genetic variants in *SNCA* are associated with PD risk, providing an important example of allelic heterogeneity (Figure 2b). Missense *SNCA* variants (i.e., p.A53T, p.A30P, p.E46K) cause autosomal dominant, familial PD, characterized by young-onset and more rapidly progressive disease (65). Such variants are exceedingly rare (frequency ≪1%), accounting for PD risk in a handful of families. By contrast, common risk variants, such as *SNCA*^*rs356168*^, are present in ~40% of European-ancestry populations and are associated with more modest effects on disease risk (odds ratio ~1.3), without readily apparent increase in family risk beyond the background rate (64). Nevertheless, both of these genetic variants affect the identical gene, *SNCA*, and point to the shared importance of αSyn-mediated disease mechanisms (66).

Other examples, such as *leucine rich repeat kinase 2* (*LRRK2*) and *glucocerebrosidase* (*GBA*), further blur the distinction between familial and sporadic PD (67–69). In both cases, incomplete-penetrance alleles were initially discovered as causes of familial PD but were also subsequently discovered in sporadic cases. Indeed, PD family history may be easily obscured by variable, age-dependent penetrance along with other potential genetic modifiers. For example, in the case of the *LRRK2*^*G2019S*^ variant, penetrance increases from 36% to 80% between the ages of 60 and 80 (70–72). *LRRK2* variants form an allelic series, including those that, compared with *LRRK2*^*G2019S*^, demonstrate either increased (p.Y1699C, p.N1437H) or decreased (p.R1441G, p.G2385R) penetrance. The latter allele, *LRRK2*^*G2385R*^, is present in approximately 5% of healthy (control) Asian-ancestry populations, causing a twofold increased risk of PD (73). Overall, while early age at onset or prior family history may increase the likelihood of genes with high-penetrance PD risk alleles, they are also unexpectedly discovered in many sporadic cases without such red flags (62, 74, 75). Even where comprehensive genetic testing is negative, it is likely that many Mendelian PD genes with incomplete-penetrance alleles remain to be elucidated, making it difficult to definitively exclude the action of such variants. Further, with more than 80 genetic loci harboring more common risk variants (frequency >1% in European-ancestry populations), genetic factors likely contribute to virtually all PD cases. In fact, even where environmental triggers are primary, such as drug-induced parkinsonism due to dopamine-blocking medications, it may be meaningful to consider the role that genetic factors play in influencing susceptibility (76). In sum, due to its complex genetic architecture, PD defies straightforward categorization into familial and nonfamilial forms.

### How Can All PD Genetic Risk Alleles Be Reliably Detected?

A remarkable spectrum of genetic variation contributes to PD risk, including both single-nucleotide variants (SNVs) and copy number variants (CNVs). Moreover, in several notable cases (e.g., *SNCA*, *PRKN*), these different categories of genetic lesions can affect the same genes, contributing to allelic heterogeneity. Besides the missense SNVs mentioned above, CNVs causing duplication or triplication of the *SNCA* locus are also associated with autosomal dominant PD (77) (Figure 2b). In addition, approximately half of pathogenic alleles in the autosomal recessive gene *Parkin* (*PRKN*) are intragenic deletions or duplications involving one or more exons (78). *PRKN* lies within an unstable fragile site of the genome that is frequently prone to rearrangements due to regions of microhomology (79), and a similar mutational mechanism appears likely for pathogenic CNVs at the *SNCA* locus (80). Even larger chromosomal deletions involving 22q11.2—which constitute the most common human microdeletion syndrome—also increase risk for PD, besides the well-known association of this structural variant with DiGeorge syndrome and schizophrenia (81). Expanding the heterogeneity of PD risk alleles even further, GGC trinucleotide repeat expansions affecting the *NOTCH2NLC* gene were recently discovered in Chinese populations (82). Repeat expansions in *NOTCH2NLC* (>66 repeats) cause neuronal intranuclear inclusion body disease, a rare neurodegenerative disorder. Whereas healthy control subjects consistently have fewer than 40 such repeats, intermediate expansions consisting of 41–64 repeats were found in 1% of PD cases. Interestingly, parkinsonism is sometimes seen in spinocerebellar ataxia type 2, which is caused by a CAG trinucleotide repeat expansion in the *SCA2* gene (83). Rarely, families with presumed autosomal dominant PD have been discovered to have *SCA2* repeat expansions, but this does not appear to be a major risk factor for sporadic PD (84). Together, *SCA2* along with *NOTCH2NLC* and the 22q11.2 microdeletion provide examples of genetic pleiotropy, in which genetic variants can cause PD along with other disease phenotypes.

The wide variety of possible genetic variants in PD—both the sheer number of genes and different types of alleles—complicates the systematic detection of responsible genetic risk factors. Currently available genetic testing methods may be insensitive for the detection of certain alleles, leading to incomplete information. SNVs are commonly detected using either genotyping arrays or sequencing approaches, which can be either targeted to specific genes or untargeted, as in whole-exome sequencing. However, exome sequencing is suboptimal for CNV detection, particularly for the smaller intragenic aberrations that are common in *PRKN* and *SNCA*. Consequently, a *PRKN*-PD case harboring a disrupting deletion in combination (*trans*-heterozygous) with a pathogenic SNV may be misdiagnosed as either homozygous or heterozygous for the SNV (80, 85). Failure to account for such CNVs may have perpetuated the hypothesis that heterozygosity for *PRKN* SNVs may increase PD risk, which appears unlikely to be true (86). CNVs are more reliably detected using alternative methods, such as array-based comparative genomic hybridization or multiplex ligation-dependent probe amplification. On the other hand, trinucleotide repeat expansions are most reliably identified using Southern blot. Nevertheless, most studies of PD genetics to date have focused exclusively on one major class of genetic variation (e.g., SNVs versus CNVs), rarely considering all possible risk alleles. Whole-genome sequencing, with its expanded coverage of exons, introns, and intergenic regions, offers the promise of capturing the full spectrum of genetic variation and may soon become the assay of choice for initial PD genetic investigations.

### Do All PD Genes Even Cause PD?

In the context of the remarkable PD clinical and pathologic heterogeneity, it can sometimes be challenging to determine the boundaries for what qualifies as a PD gene. In fact, the application of strict clinical and pathologic criteria would likely exclude many Mendelian, monogenic causes of PD and parkinsonism. Autosomal dominant and recessive forms of PD are frequently associated with extreme or atypical phenotypes; besides the prominent family history, such clinical presentations are unlikely to be mistaken for sporadic PD (87). On the basis of a systematic review of 146 *SNCA-*PD cases caused by rare SNVs or gene multiplication, the clinical picture was distinguished by young age at onset (median ~46 years), rapid progression to cognitive impairment, and frequent manifestation of other atypical features (e.g., pyramidal motor signs) (65). Pathologically, *SNCA-*PD is characterized by diffuse and severe Lewy body deposition, involving the brainstem and cortex, with sparse tau neurofibrillary tangles frequently co-occurring (58). In autosomal recessive PD caused by *PRKN*, a review of 958 cases revealed an even more extreme young age at onset (median of 31) but an overall benign disease course with rare nonmotor complications, such as cognitive impairment (78). However, as alluded to above, *PRKN*-PD lacks the characteristic Lewy body pathology, leading some to suggest that it be classified as an entirely distinct disorder along with other recessive PD syndromes (e.g., *PINK1*, *DJ-1*) (88). Notably, while most *LRRK2*-PD autopsies reveal αSyn Lewy bodies, a surprisingly wide spectrum of pathologic changes have been documented in a subset of cases, including tauopathy or, rarely, even TDP-43 protein inclusions (58).

Pleiotropy further complicates the crisp delineation of PD genes. Besides increasing PD risk, *LRRK2* alleles can rarely cause tauopathies, such as progressive supranuclear palsy, and also influence susceptibility for inflammatory bowel disease, leprosy, and cancer (89). Similarly, *GCH1* pathogenic variants, which cause the distinct, recessive disorder dopa-responsive dystonia, are associated with a significantly increased risk for PD (odds ratio ~7.5) among heterozygous carriers (90). Another intriguing example of pleiotropy stems from the discovery that *GBA* pathogenic variants causing the autosomal recessive lysosomal storage disorder Gaucher disease are among the most common genetic risk factors for PD (68, 91). Gaucher is a multisystem disorder causing heterogeneous manifestations, including enlargement of the liver and spleen and involvement of bone marrow with reduced blood counts. Rarely, more severe, neuronopathic forms of Gaucher additionally result in developmental delay or regression, seizures, and progressive neurologic decline. A link with PD was first suggested by Gaucher pedigrees including multiple heterozygous *GBA* carriers with PD (92). Subsequently, larger case-control studies confirmed that carrier states for *GBA* pathogenic variants are associated with at least a fivefold increased risk for PD (68). While such variants are uncommon in European-ancestry populations (frequency ~1%), they are more frequent in certain ethnic groups—in Ashkenazi Jews, up to 20% of PD cases are associated with *GBA* variants. Other *GBA* variants that do not cause Gaucher (e.g., p.E365K; frequency ~3% among Europeans) have also now been established as PD risk factors (93). Interestingly, beyond *GBA*, emerging evidence suggests that many other genes causing lysosomal storage disorders may also increase PD susceptibility (94, 95). In addition to *SMPD1*, which causes Niemann–Pick disease type A (96), GWAS have implicated several additional candidates, including *NEU1*, *NAGLU*, *GUSB*, *GALC*, and *progranulin* (*GRN*) (64). Remarkably, besides the recessive lysosomal storage disorder, neuronal ceroid lipofuscinosis, heterozygous loss-of-function variants in *GRN* also cause frontotemporal dementia, and common, noncoding variants that likely affect *GRN* gene expression appear to influence risk for PD, amyotrophic lateral sclerosis, and Alzheimer’s disease (97).

Pleiotropy and complex clinical phenotypes highlight the potential challenge of naming disease genes, including what is—and is not—a PD gene. Many Mendelian forms of PD adopt the *PARK* gene nomenclature [e.g., *SNCA* (*PARK1*)*, PRKN* (*PARK2*)*, ATP13A2* (*PARK9*)], but these labels have not always been applied systematically; therefore, some genes causing familial parkinsonian syndromes were initially classified using other prefixes, including for dystonias [*GCH1* (DYT5a)], ataxias [*ATXN2* (SCA2)], or neurodegeneration with brain iron accumulation [*PLA2G6* (NBIA2)]. Recent consensus statements aim to clear up some of this ambiguity in the classification of familial movement disorders, providing a useful road map (98). While embracing the value of coherent nomenclature, we nevertheless challenge the utility of relying primarily on clinical (or pathological) phenotypes. One important lesson from PD genetics has been that highly divergent and heterogeneous clinical presentations, including both familial and nonfamilial disease, often obscure remarkably similar genetic mechanisms. In short, the fundamental biology of disease may be a more robust and reliable signature for a PD gene than the distal clinical phenotype.

### Do PD Genes Act Singly or in Combination?

With approximately 100 genes implicated in PD, it is becoming clear that the relevant risk alleles are unlikely to act in isolation, potentially consistent with oligogenic mechanisms. In an early study of possible multigene models, approximately one-third of PD cases with a known monogenic cause were found to have one or more additional rare alleles for Mendelian genes causing parkinsonism (e.g., *LRRK2*^*G2019S*^ in combination with a heterozygous *ATP13A2* variant), and this additional genetic load appeared to modify disease age at onset (99). Further, in independent studies focused on *GBA* and ~50 other genes that cause lysosomal storage disorders, up to 20% of PD cases had two or more damaging variants involving distinct genes (94, 95). These results suggest the intriguing possibility of a multihit genetic model of PD in which combinatorial genetic burden in lysosomal or perhaps other biological pathways influences disease susceptibility. Currently however, a definitive test of rare-variant oligogenic mechanisms awaits larger PD sequencing datasets coupled with methodologic development for analyses of gene–gene interactions.

Meanwhile, GWAS have identified nearly 80 distinct susceptibility loci on the basis of common genetic variants (frequency 16–36%) (64). While such risk alleles individually have modest effect sizes (odds ratios ~1–2), their commonality indicates they likely act in combination. In fact, these PD risk variants are often combined to generate a genetic risk score, reflecting the aggregate risk attributable to multiple risk and/or protective alleles in one person’s genome. The genetic risk score approximates a normal distribution in European-ancestry populations, with individuals in the upper quartile having an almost fourfold increase in PD risk compared with the lowest quartile (64). Similar approaches have also been successfully utilized to capture aggregate risk due to common genetic variants affecting specific biologic pathways. For example, a polygenic score derived from variants among ~250 endolysosomal genes was significantly associated with PD risk (100). One potential limitation of most PD genetic analyses to date is that rare and common genetic variants are usually considered separately, perpetuating the rather arbitrary distinction between familial and nonfamilial disease. However, recent studies demonstrate how a genetic risk score, including all variants from GWAS, significantly modifies the penetrance of *GBA* and *LRRK2* alleles (93, 101). These important findings highlight how genetic background can influence the impact of less common and rare variant PD risk factors and may in part explain the well-established influence of race/ethnicity on the penetrance of *GBA* and *LRRK2* (71, 72). Interestingly, in a more granular, single-variant analysis of *GBA* modifiers, common regulatory variants that increase *SNCA* expression or reduce the expression of *cathepsin B* (*CTSB*), encoding another lysosomal hydrolase, were shown to significantly enhance *GBA* penetrance (93). In sum, the meaning of monogenic PD will be further diluted as we embrace the full spectrum of genomic variation underlying PD risk, such that it may soon be inappropriate to consider only the impact of single genes or variants in isolation.

### Do PD Genetic Risk Factors Actually Cause or Rather Modify Disease?

As introduced above, the symptoms of PD evolve over many decades, beginning with a protracted preclinical stage in which nervous system pathology accumulates in the absence of neurologic manifestations. Even during the prodromal stage, when signs and symptoms may first become apparent, many years may elapse before PD is clinically recognized. Consequently, in most instances it can be challenging to accurately estimate the age at onset of clinical signs, as it usually precedes diagnosis by several years. In this context, it is important to consider at what stage in the overall disease course most PD genes might act. Our conceptual model highlights several possibilities (Figure 2c). Genetic risk factors might influence the earliest formation and propagation of disease pathology (e.g., proximal causes of PD) (Figure 2c, i) or, alternatively, the downstream cascade that includes breakdown of compensatory mechanisms and ultimate clinical manifestations (e.g., conversion from preclinical to prodromal disease) (Figure 2c, ii). It is also possible that the tempo of PD progression is malleable and subject to genetic influence (Figure 2c, iii). Most genetic analyses use clinical diagnostic criteria for PD. Moreover, control subjects are not routinely screened for potential prodromal disease (e.g., RBD or reduced olfaction), let alone the possibility of clinically silent PD pathologic changes. Therefore, PD case-control genetic analyses may be more apt to detect factors influencing PD clinical manifestation and/or progression (Figure 2c, ii and iii) rather than the earliest triggers for formation and spread of Lewy body pathology (Figure 2c, i). Moreover, since these study designs aggregate all PD cases together to achieve maximal power for discovery, they necessarily ignore the remarkable phenotypic variation between cases. Indeed, beyond PD initiation and progression, genetic factors might plausibly impact its heterogeneous manifestations, such as the presence and severity of specific motor and nonmotor features (Figure 2c, iv).

Recent analyses have begun to address these questions by leveraging more granular information on disease phenotypes, beyond case-control status. For example, in *LRRK2*-PD, several studies have suggested an overall more benign disease course, while others reveal a higher likelihood of postural instability and gait impairment (70, 102). Additionally, in the case of *GBA*, evidence has emerged to support an allelic and phenotypic series. Specifically, mutations causing more significant reduction in glucocerebrosidase enzymatic activity and more severe, neuronopathic Gaucher consistently result not only in greater PD penetrance (among heterozygous carriers) but also in decreased age at onset, more rapid decline, and increased dementia risk (103, 104). Similarly, in a GWAS including more than 28,000 PD cases, it was found that most of the common genetic variants that were previously linked to increased PD risk, including *GBA*, *SNCA*, and *TMEM175*, along with a multilocus genetic risk score, were also consistently associated with PD age at onset (105). Despite a well-powered analysis, there were several notable exceptions, including the *microtubule associated protein tau* (*MAPT*) locus, which is one of the strongest PD susceptibility signals from GWAS. This unexpected result highlights that a subset of PD risk alleles may act via a divergent genetic mechanism, in which disease risk is decoupled from age at onset. While it is challenging to fit this observation within our conceptual model (Figure 2c), it may suggest that some loci such as *MAPT* may act at a much earlier, developmental stage in the overall pathophysiologic cascade than most other risk factors. In the coming years, larger cohorts with greater depth of phenotyping will likely permit even more powerful genetic dissection of PD heterogeneity.

As introduced above, one important contributor to PD heterogeneity is the co-occurrence of other age-related brain pathologies, prompting the hypothesis that PD genetic risk might overlap with that of other neurodegenerative disorders. Consistent with this hypothesis, common genetic variation at the *apolipoprotein E* (*APOE*) locus that increases risk for Alzheimer’s disease has also been consistently and robustly associated with PD age at onset and the development of PD dementia (106, 107). Alternatively, it is possible that *APOE* influences αSyn pathology independent of Alzheimer’s pathology (108, 109). A genetic risk score incorporating dozens of Alzheimer’s disease susceptibility loci was also significantly associated with susceptibility for Lewy body dementia in a large meta-analysis including more than 2,500 cases (110). However, in genome-wide analyses examining correlation of heritability due to common genetic variation, there was little suggestion for deeper allele sharing between Alzheimer’s disease and PD (111). Overall, these results support a model in which genetic risk for Alzheimer’s disease either accelerates PD progression (Figure 2c, iii) or otherwise alters the trajectory of disease toward distinct phenotypic subtypes, such as the diffuse malignant form of PD (Figure 2c, iv).

## MECHANISMS

Despite the enormous progress highlighted above, the discovery of a genetic variant is only the first step to understanding its impact on PD pathogenesis. In each case, we must next answer (*a*) how disease-associated variants alter gene function and (*b*) how these genes impact disease mechanisms. Answering the first question is complicated by the fact that most variants implicated by GWAS fall into intronic or intergenic sequences, implicating extensive genomic regions and a large number of potential candidate genes. From the latest PD GWAS, more than 305 candidate genes can be nominated from 78 distinct genomic loci (64). Despite the presence of a compelling candidate, even the *SNCA* genomic locus is complex, harboring multiple, independent common variant associations with PD risk (64). One of these, a risk variant affecting intron 4 enhancer sequences, was experimentally validated to increase *SNCA* expression, on the basis of gene-editing studies in human neuronal cultures (112). In other cases, the availability of large-scale human reference transcriptomes and epigenomes has significantly improved our ability to prioritize candidate genes for further study. Nevertheless, definitive identification of the causal variants and responsible gene(s) for common variant susceptibility signals remains a significant, unsolved challenge in most cases (see the sidebar titled Functional Genomics of the *MAPT* Locus). In contrast to most GWAS-defined susceptibility loci, rare variant analyses in families or large population samples may be more apt to precisely define the likely causal genes and thereby facilitate mechanistic follow-up. However, while some variants are obviously damaging (e.g., nonsense or frameshifting mutations), many others cause nonsynonymous amino acid changes of uncertain significance, requiring experimental studies to confirm pathogenicity. For example, rare, high-penetrance missense alleles of *SNCA* were shown to alter protein folding, propensity for oligomerization, and a wide variety of other functional consequences that implicate toxic mechanisms (66).

Beyond pinpointing the responsible genes, we also face significant obstacles in the elucidation of pertinent PD genetic mechanisms: (*a*) many emerging candidate genes have unknown functions, especially within the nervous system; (*b*) understanding neurodegeneration requires genetic analysis within the context of the aging, adult brain; (*c*) gene functions must often be considered separately in the context of heterogeneous brain cell types (e.g., neurons, neuronal sub-types, glia); and (*d*) since many genes likely confer risk in combination, simultaneous experimental manipulation of multiple genes may be required. The field has nevertheless made remarkable progress in unraveling the biology of Parkinson’s syndrome (113, 114), with various animal models playing an important role (see the sidebar titled Animal Models for PD). Whereas heterogeneous clinical, pathologic, and genetic features might suggest myriad and fragmented mechanisms, there has instead been an emerging consensus. Results so far have converged to highlight several over-lapping biological themes, including important roles for synaptic, lysosomal, mitochondrial, and immune pathways. While much work yet remains, the discovery of coherent biological pathways underlying the pathogenesis of PD—along with other atypical parkinsonian syndromes—offers hope for the development of targeted therapies with potential broad applicability.

### Synapse

**Figure 3:**
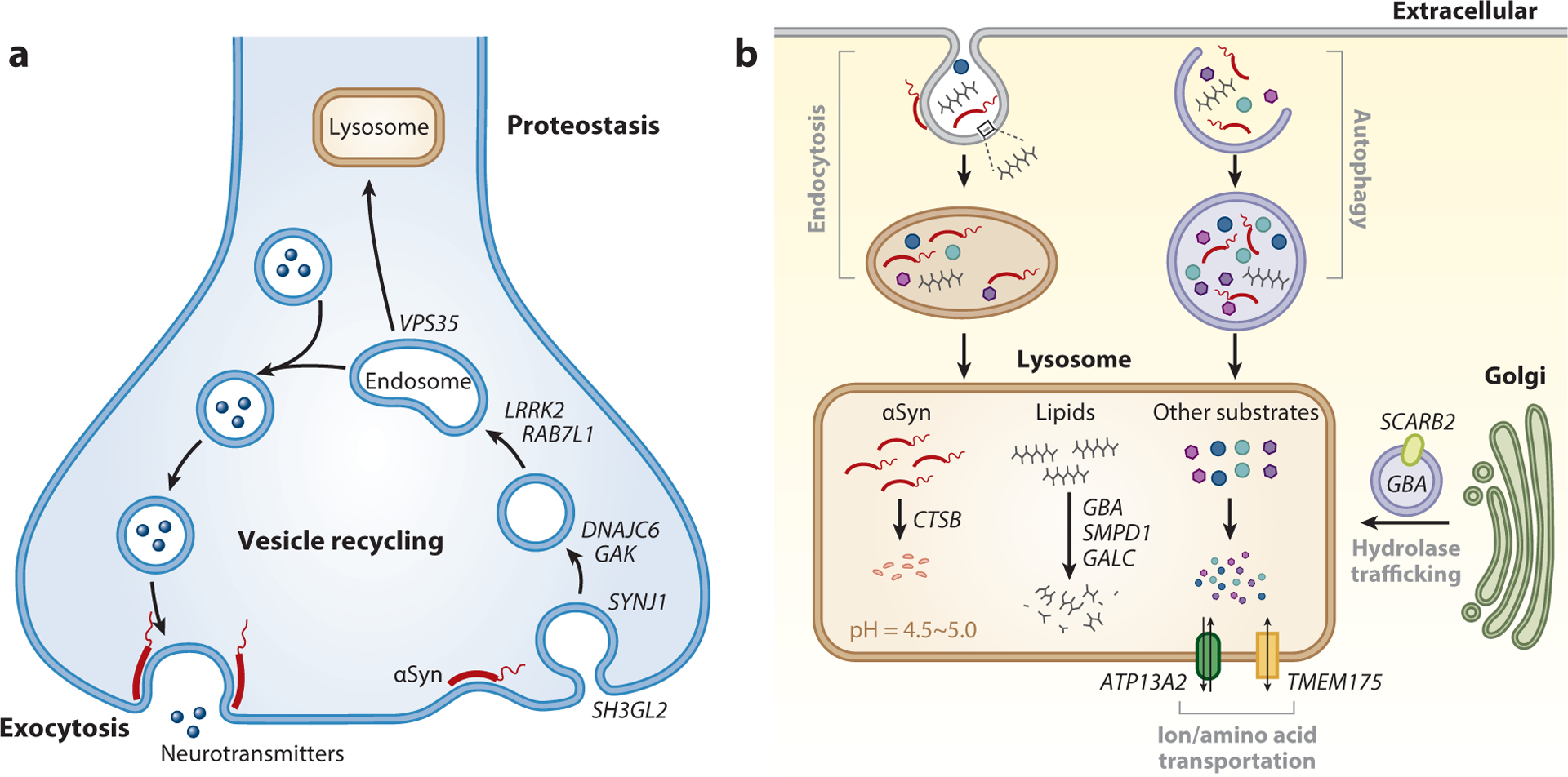
Parkinson’s disease (PD) genes implicate synaptic and lysosomal mechanisms. (*a*) The synapse. Synaptic transmission is initiated when a presynaptic nerve terminal releases neurotransmitters, via exocytosis, into the synaptic cleft, resulting in a response in the postsynaptic cell. Synaptic vesicles are recycled by clathrin-mediated endocytosis and refilled with neurotransmitters. *DNAJC6*, *SYNJ1*, *GAK*, and *SH3GL2* participate in clathrin-coated vesicle endocytosis and recycling, whereas *VPS35*, *LRRK2*, and *RAB7L1* modulate endolysosomal trafficking. Under physiological conditions, alpha-synuclein (αSyn) regulates neurotransmitter release possibly via pleiotropic actions, including endocytosis, synaptic vesicle clustering, and exocytosis. (*b*) The lysosome. αSyn reaches the lysosome either through endocytosis or autophagy. Membrane lipids are also trafficked within the endolysosomal system. Many PD genes encode lysosomal proteins involved in the degradation of lysosomal contents (*CTSB*) and membrane lipids (*GBA*, *SMPD1*, *GALC*), the proper trafficking of lysosomal enzymes (*SCARB2*), and the maintenance of the compartmental milieu (*ATP13A2*, *TMEM175*).

Synaptic dysfunction and loss are early pathologic correlates in PD (23), and numerous PD genes have been implicated in synaptic transmission and/or synaptic vesicle recycling pathways (Figure 3a). In particular, αSyn has been linked to multiple steps in neurotransmission, including synaptic vesicle clustering, exocytosis, and recycling via clathrin-mediated endocytosis (30). The N-terminal amphipathic, α-helical domain of αSyn interacts with phospholipids and alters plasma membrane fluidity and curvature, with potential consequences for synapse structure and dynamics (115). In addition, the C-terminal acidic domain directly binds synaptobrevin-2, promoting SNARE complex assembly during exocytosis (116). Nevertheless, loss-of-function studies have yielded equivocal results on the specific requirements for αSyn at the synapse, and many other potential cellular targets have also been identified. Further, the *SNCA* gene is not present in invertebrates, suggesting that rather than being part of conserved core synaptic machinery, αSyn subserves a more ancillary, regulatory role. One unsolved puzzle is the nature of the relationship between the physiologic, normal function of αSyn at the synapse and its contribution to PD pathogenesis. On the basis of systematic mutagenesis, most sequence changes either selectively promote αSyn aggregation and toxicity or disrupt its physiologic functions (117). Indeed, substantial evidence supports gain-of-function, toxic mechanisms in PD, either due to (*a*) common *SNCA* regulatory polymorphisms that enhance gene promoter activity, (*b*) similarly increased expression due to locus multiplication, or (*c*) rare variants that promote aggregation. Familial PD mutations cluster adjacent to the αSyn hydrophobic central core, called the nonamyloid β component, which together with immediately flanking sequences drive protein fibrillization (30, 118). A gain-of-function mechanistic model is further supported by numerous αSyn transgenic models, in which overexpression and/or introduction of disease mutations recapitulate many PD pathologic features, including early and progressive disruption of synaptic structure and function (66).

Follow-up studies of the *LRRK2* gene also strongly implicate synaptic mechanisms. LRRK2 is a large (2,527 amino acids) cytosolic protein including both kinase and GTPase catalytic sites, along with multiple other protein–protein interaction domains. LRRK2 is present at the synapse and associates with and/or phosphorylates many other proteins involved in vesicle endocytosis and trafficking (113, 119). Moreover, loss-of-function studies from multiple animal models reveal conserved requirements for *LRRK2* in synaptic vesicle endocytosis and neurotransmission (120, 121). Critically, many LRRK2 protein interactors and substrates have been independently identified as PD gene candidates from GWAS, including *SH3GL2* (also called *endophilin-A1*), *cyclin-G-dependent kinase* (*GAK*), and *RAB7L1* (64). While these genes have yet to be definitively proven as causal, they are nevertheless compelling guilt-by-association candidates on the basis of interactions with LRRK2 and coparticipation in synaptic vesicle endocytosis (120–122). Genes identified from Mendelian recessive, young-onset, parkinsonian syndromes, including *synaptojanin 1* (*SYNJ1*) and *DNAJC6* (also called *auxilin*), further reinforce the importance of synaptic mechanisms and solidify connections between familial and sporadic forms of PD (123, 124). SYNJ1 is a target of LRRK2 kinase activity and also directly interacts with SH3GL2 during synaptic vesicle endocytosis (125, 126). DNAJC6 is a paralog of GAK, and these closely-related proteins similarly associate with LRRK2 and participate in the removal of synaptic vesicle clathrin coats (127). Lastly, experiments in both mammalian neurons and *Drosophila* models have revealed conserved gene–gene interactions among *LRRK2*, *RAB7L1*, and *VPS35*, which is a rare cause of autosomal dominant, late-onset PD (128–130). *VPS35* encodes a core component of the retromer protein complex, which also participates in synaptic transmission, besides its established role in endosome-lysosomal trafficking (131, 132). Overall, these results support a dense synaptic susceptibility network underlying PD pathogenesis (Figure 3a).

Several *LRRK2* variants have been shown to enhance kinase activity (73), consistent with a toxic gain-of-function mechanism, and increased LRRK2 activity has also been implicated in postmortem brain tissue from idiopathic PD cases lacking *LRRK2* mutations (133). By contrast, the recessive PD genes, such as *SYNJ1* and *DNAJC6*, unequivocally act via loss-of-function mechanisms. Intriguingly, many of these PD genes with synaptic functions have also been linked to synuclein-mediated disease mechanisms. Specifically, knockdown of *SYNJ1*, *GAK*, and *VPS35* independently enhanced αSyn-induced neurodegeneration, as shown in studies in multiple transgenic models, including yeast, *Drosophila*, and mice (134–137). Moreover, in mammalian neurons, proximity labeling coupled with mass spectrometry further highlights close associations between αSyn and most other synaptic PD genes (138). Overall, a hybrid gain- and loss-of-function model seems probable, in which toxic changes in αSyn and/or LRRK2 may comprise a key early trigger, but the subsequent neurodegenerative cascade, including downstream targets, is influenced by synaptic localization and interaction partners. The resulting synaptic degeneration may thus influence the transition from the preclinical to the prodromal stage of disease (Figure 2c, ii).

### Lysosome

The lysosome is a hub for cellular homeostasis, including turnover of proteins, lipids, and other macromolecules, via endocytosis, phagocytosis, or autophagy (139). A remarkable number of PD genes encode lysosomal enzymes (e.g., *GBA*), lysosomal membrane proteins (e.g., *TMEM175*), or regulators of endosomal-lysosomal trafficking (e.g., *LRRK2*, *VPS35*) (Figure 3b). Critically, the autophagy-lysosomal pathway contributes to αSyn proteostasis (140, 141). Therefore, lysosomal dysfunction can hinder αSyn clearance, promoting its aggregation, pathologic propagation, and cytotoxicity (142). Reciprocally, as demonstrated in independent studies across numerous cellular and animal models, toxic αSyn species disrupt lysosomal biogenesis and function, promoting a positive feedback loop that may be a key driver for disease pathogenesis (143–145).

The discovery of *GBA* loss-of-function variants as common and potent risk factors has significantly accelerated our understanding of PD lysosomal mechanisms (68, 91). *GBA* encodes the lysosomal enzyme, glucocerebrosidase, which hydrolyzes the sphingolipid, glucosylceramide, to ceramide and glucose. Glucocerebrosidase activity may also be reduced in idiopathic PD among individuals without *GBA* variants (146, 147). *GBA* loss of function can alter αSyn accumulation and/or aggregation (148, 149), possibly due to the buildup of glucosylceramide and glucosylsphingosine (150, 151) or via disruption of autophagy (152). Besides *GBA*, several other PD gene candidates, including *SMPD1* and *GALC* (encoding acid sphingomyelinase and galactosylceramidase, respectively), are similarly involved in sphingolipid metabolism, and *SCARB2* encodes the receptor responsible for trafficking GBA to the lysosome (64, 153). In addition, *CTSB* encodes a lysosomal cysteine protease that directly participates in the lysosomal degradation of αSyn (154), providing a mechanism for how variants at this locus may increase PD risk and further modify *GBA* penetrance (93).

Lysosomal membrane proteins include a diversity of channels and transporters that have an important regulatory role in maintaining the compartmental milieu (139). *TMEM175* encodes one such protein that regulates lysosomal membrane potential and pH (155, 156) and further provides an unusual example of a gene nominated by PD GWAS that was successfully fine-mapped to pinpoint two independent missense alleles responsible for the association. Remarkably, the two variants, p.M393T and p.Q65P, cause loss or gain of function, respectively, with opposing effects on PD risk or protection (157, 158). In cell culture, *TMEM175* knockdown attenuates both CTSB and GBA enzymatic activity and promotes αSyn accumulation (159, 160). Moreover, in mice, *TMEM175* loss of function causes dopaminergic neuronal loss and progressive motor impairment (160). Another lysosomal transporter gene, *ATP13A2*, is the cause of Kufor–Rakeb syndrome, a recessive, young-onset, rapidly progressive parkinsonian dementia (161). Recent studies establish ATP13A2 as a lysosomal exporter of polyamines that has roles in scavenging heavy metals and reactive oxygen species (162). Furthermore, experiments across multiple cellular and animal models highlight that knockdown or overexpression of *ATP13A2* reciprocally enhances or suppresses αSyn toxicity (163).

In conclusion, human genetic and functional evidence strongly implicate lysosomal mechanisms in PD susceptibility. Beyond triggering the onset and earliest deposition of αSyn pathology (preclinical PD, Figure 2c, i), ample evidence supports a vicious cycle with potential to accelerate PD progression (Figure 2c, iii). Since impaired autophagic flux and reduced proteostasis are hallmarks of aging (164), the lysosome may also mediate interactions between implicated genetic variants and aging, affecting PD risk. Lastly, progressive failure in endolysosomal function is predicted to negatively impact many other cellular targets, including other core pathways in PD pathogenesis, namely the synapse and mitochondria (131, 165, 166).

### Mitochondria

The first clues of an important link between mitochondria and PD pathogenesis came from an outbreak of methyl-phenyl-tetrahydropyridine (MPTP)-induced parkinsonism among a cluster of heroin users in California during the 1980s (167). MPTP and similar mitochondrial toxins were shown to trigger dopaminergic neuronal loss, and these agents continue to be used for a variety of PD animal models (114). A succession of genetic discoveries, particularly in Mendelian forms of parkinsonism, along with intensive experimental follow-up, have strongly reinforced the role of mitochondrial biology in PD risk (113, 168). Pioneering work in *Drosophila* models revealed that conserved homologs of the recessive PD genes *PRKN* and *PINK1* function coordinately in mitochondrial quality control (169, 170). Subsequent studies demonstrated that PINK1, a serine-threonine kinase, can detect damaged mitochondria and recruits PRKN, an E3 ubiquitin ligase, which subsequently triggers lysosomal autophagy (mitophagy) (171). Additionally, *CHCHD2*, a rare cause of autosomal dominant parkinsonism, also encodes a mitochondrial protein and appears to respond to mitochondrial insults in a *PRKN/PINK1-*dependent manner (172–174).

Compared with rare genetic variants causing Mendelian PD, human genetic studies suggest a more limited role for common genetic variation among mitochondrial genes in nonfamilial PD (175). One notable exception is *VPS13C*, which was initially discovered as a cause of recessive early onset PD and was subsequently highlighted as a candidate gene from PD GWAS (64, 176). In human cellular models, silencing *VPS13C* altered mitochondrial morphology, reduced mitochondrial membrane potential, and increased PINK1/PRKN-dependent mitophagy (176, 177). Importantly, knockdown of a conserved *VPS13C* ortholog in *Drosophila* also enhanced αSyn-induced neurodegeneration. Indeed, substantial evidence supports a model in which toxic αSyn species can target mitochondria, and mitochondrial PD genes subsequently either accelerate or compensate for αSyn-mediated disease mechanisms (30, 178). For example, in a recent proof-of-principle therapeutic study, PRKN rescued αSyn-mediated mitochondrial damage in multiple cellular and mouse PD models (179). Further supporting mechanistic cross talk, experimental manipulations of other PD genes with primary roles in endolysosomal trafficking, including *LRRK2* and *VPS35*, also impact mitochondrial dynamics and quality control (178). Reciprocally, mitochondria critically support the energy-intensive physiology of synaptic transmission (180). In sum, we propose that PD genes participating in mitochondrial mechanisms likely function as downstream mediators of αSyn pathology, possibly during the prodromal or later stages of disease (Figure 2c, ii). In addition, the relative selectivity of *PRKN*-PD for substantia nigra degeneration (58), along with the oxidative milieu of dopaminergic neurons (181), supports a plausible secondary role for mitochondrial PD genes in cell-type vulnerability.

### Immune Response

PD GWAS identify a strong PD susceptibility signal overlying the human leukocyte antigen region on chromosome 6, which encodes multiple proteins composing the major histocompatibility complex (MHC) class II that mediates antigen presentation (64). MHC-II alleles associated with PD can preferentially display peptides derived from αSyn and drive T cell responses, potentially contributing to PD pathogenesis (182). Several other PD gene candidates are also expressed in microglia, and PD risk alleles at these loci (e.g., *P2RY12*) are associated with altered gene expression (183). These results suggest that changes in microglial gene expression may mediate the associations of such loci with PD. Microglia have also been implicated in the uptake, turnover, and/or propagation of αSyn aggregates (184, 185). Indeed, many PD genes involved in neuronal endolysosomal biology (e.g., *LRRK2*) could impact microglial phagocytosis and function. A recent study using a mouse model further links the recessive PD gene *PINK1* to mitochondrial antigen presentation and immune-mediated dopaminergic neuronal loss, and this cascade was triggered by intestinal bacterial infection (186). Notably, microglial and innate immune mechanisms have also been strongly implicated in Alzheimer’s disease, including downstream of *APOE*, an important PD modifying gene (106, 107). Compared with Alzheimer’s disease, however, the relative enrichment for PD genes among microglial expression data is less pronounced, perhaps consistent with a supporting role. Overall, genetic and experimental evidence support an emerging role for immune mechanisms in PD risk, perhaps as a hub for gene–environment interactions.

## CONCLUSIONS

A little more than two decades ago, PD was considered by most to be environmentally triggered, with minimal support for a genetic etiology. Today, we know of ~100 PD risk loci, and these insights have directly spurred clinical trials targeting *SNCA*, *LRRK2*, and *GBA*, among other genes (187). Besides identifying patients who will benefit from these emerging therapies, genetics may soon offer personalized predictions on heterogeneous features that impact disease-related disability (e.g., dementia risk or PD subtypes). Remarkably, genetic testing or counseling is still rarely pursued in routine clinical practice, despite strong interest from most patients and families in learning their genetic information (188). Major research initiatives are beginning to fill the void, offering genetic testing to many patients. In the coming years, it will be essential to ensure that all can benefit from genetic advances. Whereas most studies have previously focused on white, European-ancestry subjects, limiting generalizability, many current efforts are embracing diversity and inclusion.

Parkinson’s syndrome is clinically, pathologically, and genetically heterogeneous. It is possible that each patient represents a virtually unique, forme fruste of disease shaped by a combination of genetic and nongenetic factors. Thus, rather than a single disorder, PD may instead encompass hundreds of different diseases. Precision medicine—the promise of more individualized diagnoses and targeted therapies—is thus particularly suited to PD. Given the modest risk and variable penetrance for most implicated variants, genetic testing will need to be combined with other biomarkers (e.g., sleep or olfactory testing, neuroimaging) to achieve useful predictive and/or diagnostic power (5). Since PD develops over decades, it will be important to map the individual genes to specific stages in the overall pathogenic cascade (Figure 2c). PD genetic insights and follow-up mechanistic dissection are revealing unexpected links between genes and across disparate parkinsonian disorders. One critical need is to develop improved biomarkers to detect and monitor pathologic progression, including for implicated genetic pathways (e.g., lysosomal or synaptic dysfunction). We are thus approaching an inflection point where clinical syndromic diagnosis may yield to more direct assessment of molecular drivers, enabling more tailored and effective interventions.

## FUNCTIONAL GENOMICS OF THE *MAPT* LOCUS

Chromosome 17q21 harbors a 900-kb chromosomal inversion defining a common genetic variant haplotype (frequency ~70%) called H1 that is strongly associated with susceptibility for PD as well as the atypical parkinsonian disorders progressive supranuclear palsy and corticobasal degeneration (189). This genomic region includes *microtubule associated protein tau* (*MAPT*) along with other potential candidate genes, leading to intensive efforts to pinpoint the causal gene(s) and variant(s). While analyses of bulk brain tissue RNA sequencing link the H1 haplotype to increased *MAPT* expression and/or alternative splicing (190), other work focusing on gene expression changes in dopaminergic neurons pinpoints *KANSL1* as a potential mediator (191). More recently, a specific H1 variant disrupting a *MAPT* ribosome binding site was nominated as a putative causal variant, highlighting how in addition to its potential transcriptional consequences, MAPT protein translation and proteostasis may also be affected (192). Studies in PD experimental models show that aggregated tau species may potentiate αSyn aggregation and toxicity (32, 193). Besides H1, more than 50 rare variants have been discovered to alter *MAPT* alternative splicing or other proteotoxic mechanisms, but these alleles cause autosomal dominant frontotemporal dementia with parkinsonism, a tauopathy, which differs both clinically and pathologically from PD (17).

## ANIMAL MODELS FOR PD

Model organisms, including mice (194, 195), fruit flies (196, 197), nematodes (135, 177), and yeast (145, 198), have been essential in the dissection of PD genetic mechanisms. Models based on *SNCA* variably recapitulate salient clinical and pathologic features of PD (e.g., dopaminergic neuron loss, locomotor impairment, and the formation/propagation of Lewy body–like αSyn aggregates). Genetic models have also enabled high-throughput screening strategies for rapid follow-up of large candidate gene lists from PD human genetics or identification of promising new therapeutic targets (134, 177, 199). Besides mechanistic follow-up of PD genes, models are powerful tools for confirming pathogenicity of implicated variants of uncertain significance. More recently, induced pluripotent stem cell–derived dopaminergic neuronal cultures and midbrain-like organoids have emerged as complementary strategies for PD functional genomics (181).

## Abbreviations

Parkinsonism: pattern of motor impairment (movement speed, muscle tone, tremor, gait, postural reflexes) caused by Parkinson’s disease and other disorders that similarly disrupt basal ganglia function
Allelic heterogeneity: when multiple independent variants affecting the same gene cause a common phenotype (such as Parkinson’s disease)
Penetrance: the likelihood that a person with a genetic risk variant will develop Parkinson’s disease
Sporadic PD: cases without a known family history or an apparent genetic risk of Parkinson’s disease (PD); sometimes used interchangeably with the term idiopathic PD
Familial PD: cases with a positive family history of Parkinson’s disease (PD); strong familiality sometimes indicates monogenic or Mendelian PD due to dominant or recessive inheritance
Genome-wide association studies (GWAS): a method to discover risk alleles by comparing genetic variants sampled from across the genome for different frequencies between cases and controls; usually applies to common variants (polymorphisms)
Single-nucleotide variants (SNVs): changes affecting one specific nucleotide in the genome; common SNVs (frequency >1%) are often called single-nucleotide polymorphisms (SNPs)
Copy number variants (CNVs): structural changes in the genome that can range in size and type, including deletions and duplications
Oligogenic: multiple genes acting together to influence disease risk; when more than a few genes are involved, the term polygenic is often used
